# Quantification of the amount of surface groups of aminated silica nano- and microparticles utilizing a fluorine tag and HR-CS-GFMAS

**DOI:** 10.1007/s00216-025-06107-4

**Published:** 2025-09-22

**Authors:** Isabella Tavernaro, Fabian Simon, Lennart Gehrenkemper, Charlie Tobias, Ute Resch-Genger, Björn Meermann

**Affiliations:** 1 Division 1.1 - Inorganic Trace Analysis (ITALab), Federal Institute for Materials Research and Testing (BAM), 12489 Berlin, Germany; 2 Division 1.2 - Biophotonics, Federal Institute for Materials Research and Testing (BAM), 12489 Berlin, Germany; 3 Department of Chemistry, Georgetown University, Washington, D.C., USA

**Keywords:** Surface functional group quantification, Fluorine tags, Silica nano- and microparticles, Optical assays, Potentiometric back titration, High resolution-continuum source-graphite furnace molecular absorption spectrometry (HR-CS-GFMAS)

## Abstract

The performance, bioavailability, and safe use of engineered nanomaterials (NMs) depends not only on properties such as size, shape, and surface area, but largely on surface chemistry. While many sizing methods have been established, there is still a lack of validated screening methods for determining NM surface functional groups (FGs). In this context, we present a fast and simple method for FG quantification using high resolution-continuum source-graphite furnace molecular absorption spectrometry (HR-CS-GFMAS) and assess its applicability for the surface analysis of representatively chosen aminated silica nanoparticles (NPs) and microparticles (MPs) in conjunction with amino FG labeling with a fluorine tag. For this proof-of-concept study, first surface amino FG screening of the silica NPs and MPs was done with a potentiometric back titration method, providing the total amount of protonatable surface FGs, and two optical assays relying on reporter dyes with sizes and spatial requirements, i.e., surface binding areas smaller or larger than that of the fluorine tag to estimate the maximum and reporter-accessible number of amino FGs. Subsequently, the surface amino FGs were labeled with the fluorine tag 4-(trifluoromethyl)benzoic acid (TFMB) and the amount of fluorine originating from the bound TFMB molecules was quantified by HR-CS-GFMAS in two common organic solvents, i.e., dimethyl sulfoxide (DMSO) or ethanol (EtOH) to assess possible interferences from organic matrices. Our study revealed limits of detection (LODs) and quantification (LOQs) for fluorine of 1.0 µg/L and 3.5 µg/L in EtOH and 1.5 µg/L and 5.0 µg/L in DMSO, respectively. Overall, a quick and simple method for analyzing surface FGs on NPs and MPs was presented utilizing broadly available fluorine tags and HR-CS-GFMAS for fluorine quantification, which can be applied, e.g., for homogeneity, stability, and aging studies of surface-modified particles. This could contribute to ease the understanding of property-safety relationships for surface-functionalized NMs.

**Graphical Abstract:**

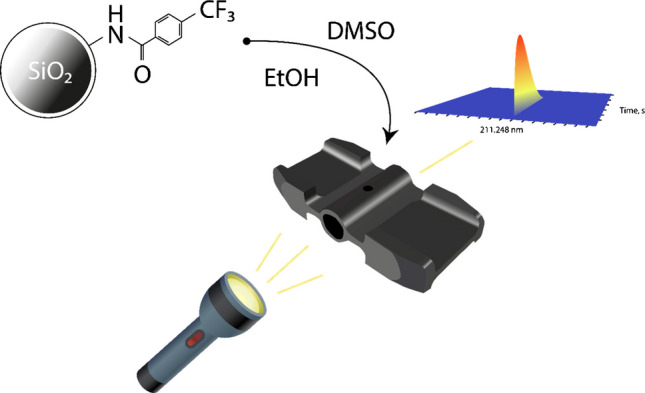

## Introduction

Over the past few years, fluorine-modified nanomaterials, increasingly used, e.g., in biomedical imaging, have raised significant interest regarding their environmental fate and transport studies, functional material design, and analytical method development [1–5]. The performance and safety of such fluorine-modified nanomaterials depend not only on their size and shape, but largely on their surface chemistry and surface FGs [6–8], which determines their chemical reactivity, environmental behavior, and interactions with biological species. This requires sensitive and selective methods for fluorine detection and quantification [9]. Methods applied for fluorine analysis range from simple sum parameter methods such as elemental analysis [10, 11] over chromatographic separation methods like gas chromatography-mass spectrometry (GC-MS) or pyrolysis-GC/MS [12], [13] to X-ray photoelectron spectroscopy (XPS) [14–16] and 19-fluorine nuclear magnetic resonance spectroscopy (^19^F NMR) [17, 18]. Particularly, the latter two methods are also utilized for surface group analysis on nanomaterials (NMs) [8] and are utilized for the labeling of surface FGs with fluorine-containing tags bearing reactive groups for their site-specific covalent attachment [19]. Although fluorine tags help to enhance the sensitivity of these two techniques, XPS faces challenges in quantification due to its limited effective probing depth of about 5 nm, the need to consider particle size-dependent curvature effects by suitable simulation, and the considerable influence of sample preparation on the accuracy and uncertainty of measurement results [8, 15, 20, 21], while ^19^F NMR, applicable to both solid and liquid samples, demands relatively large sample amounts and has a relatively low sensitivity in the lower ppm region [22–24]. In addition, both methods require sophisticated and relatively expensive instrumentation and experienced operators for the performance of the measurements and data analysis. FGs on nano- and microparticles can also be quantified by broadly available atomic absorption spectrometric (AAS) techniques. This has been demonstrated, e.g., for the analysis of metals and metalloids [25]. Since several years, high resolution-continuum source-atomic absorption spectrometers are available on the market, which enable the analysis of halogens via molecular absorption (HR-CS-GFMAS). In HR-CS-GFMAS, diatomic metal-halogen species are formed in situ, offering high sensitivity in the lower ppb range. HR-CS-GFMAS is particularly sensitive for fluorine when gallium is applied as a modifier, leading to gallium-monofluoride [26–28]. Currently, this technique is primarily used for the analysis of per- and polyfluorinated alkyl substances (PFASs) in environmental samples [28–31].

The high sensitivity of HR-CS-GFMAS in combination with the relatively simple instrumentation and ease of use together with the broad availability of different types of fluorine tags renders this analytical method attractive for the analysis and quantification of FGs on nanoparticles (NPs) and microparticles (MPs). This encouraged us to assess the applicability of a straightforward HR-CS-GFMAS method for quantifying amino FGs, which are among the most broadly surface FGs on different types of NMs, on aminated silica NPs and MPs dispersed in different organic solvents, utilizing 4-(trifluoromethyl)-benzoic acid (TFMB) to quantitatively label these surface amino FGs. Aminated silica NPs and MPs were chosen as exemplary particle systems due to their broad usage in the life and material sciences and in consumer products. Within the context of method development, we examined possible interferences originating from organic solvents in the measured complex molecular spectra, as organic solvents like ethanol (EtOH), dimethyl sulfoxide (DMSO), *N*,*N′*-dimethylformamide (DMF), and toluene are often utilized for dispersing and storing NMs. As prerequisites to judge the results obtained by our proof-of-concept study with HR-CS-GFMAS and a fluorine tag, we performed potentiometric back titrations with the aminated silica particles, providing the total amount of protonatable FGs, which commonly matches with the total number of surface amino FGs, and two optical assays, which both include a labeling step with an optical reporter for signal generation and yield the amount of surface amino FGs accessible for the respective reporter. As measures to estimate the amount of surface amino FGs per particle that can be covalently labeled with TFMB molecules, subsequently quantified by HR-CS-GFMAS, we chose assays utilizing optical reporters of smaller and larger size and spatial demand than TFMB. Thereby, these method cross-comparisons could be used to judge the reliability and performance of our HR-CS-GFMAS method relying on FG labeling with a fluorine tag.

## Experimental

### Syntheses of fluorine tag-modified silica nanoparticles (SiO_2_-NPs) and silica microparticles (SiO_2_-MPs)

SiO_2_-NPs and SiO_2_-MPs were synthesized in a mixture of ethanol (EtOH, absolute, 99.9%, ChemSolute, Th. Geyer) and ultrapure water (0.055 μS∙m^−1^, Milli-Q water, produced using a Milli-Q® Advantage A10 System (Merck KGaA)) according to the well-known Stöber sol-gel approach using ammonia (NH_4_OH, 25%, abcr GmbH) as a catalyst and tetraethyl orthosilicate (TEOS, 99%, Sigma-Aldrich) as the silicon precursor [32, 33]. SiO_2_-NPs were prepared as described in the literature [34] by varying the ratio of EtOH, water, NH_4_OH, and TEOS. For the synthesis of SiO_2_-MPs, 0.32 mmol (23.8 mg) potassium chloride (Sigma-Aldrich), 75 mL of EtOH, 9.5 mL of Milli-Q water, 34 mL of ammonia, and 1.55 mL of TEOS were mixed and stirred at 40 °C. Next, 4.34 mL of TEOS dissolved in 26 mL of EtOH was added dropwise to the reaction mixture over a period of 2 h. After the addition of TEOS, the particle dispersion was stirred for an additional 15 h to finalize the particle growth. The obtained particles were purified by centrifugation (11,000 rcf, 20 min) and washed thrice with an EtOH/water mixture.

In a post-synthesis step, the particle surface was modified with 3-(aminopropyl)triethoxysilane (APTES, abcr GmbH) to introduce amino FGs [35]. Therefore, the unmodified SiO_2_-NPs and SiO_2_-MPs were redispersed in 20 mL of EtOH and stirred at 30 °C under an argon atmosphere for 30 min before an excess of APTES was added. The reaction mixture was stirred for an additional 48 h under a protecting gas atmosphere, followed by centrifugation (11,000 rcf, 20 min) and three washing steps with EtOH. Finally, the amino surface FGs of the aminated SiO_2_-MPs and SiO_2_-NPs were functionalized with 4-(trifluoromethyl)benzoic acid (TFMB, 98%, Sigma-Aldrich) as a prerequisite for surface amino FG quantification with HR-CS-GFMAS. 20 mg of the aminated silica particles were redispersed in 5 mL of toluene (anhydrous, 99.8% Sigma-Aldrich) and stirred under an argon atmosphere at 40° C for 20 min. Next, 3.05 µmol (SiO_2_-MPs) or 0.14 mmol (SiO_2_-NPs) of 1-ethyl-3-(3-dimethylaminopropyl)carbodiimide (EDC, ≥ 98.0% Sigma-Aldrich) and *N*-hydroxy-succinimide (NHS, 98% Sigma-Aldrich) in 1 mL of toluene, respectively, were added to the reaction mixture and stirred for 1 h. Then, 4.16 µmol (SiO_2_-MPs) or 0.20 mmol (SiO_2_-NPs) of TFMB in 1 mL of toluene were added and stirred under an argon atmosphere at 40 °C overnight. To purify the fluorine tag-labeled silica particles, the reaction mixtures were centrifuged (11,000 rcf, 20 min) and washed several times to remove unreacted TFMB and the coupling reagents. The particles were then dried and redispersed in EtOH or DMSO.

### Particle characterization

The physicochemical properties of the silica NPs and MPs were characterized by transmission electron microscopy (TEM), dynamic light scattering (DLS), nano tracking analysis (NTA), and zeta potential measurements. Particle morphology, state of agglomeration, and number-based average particle diameter were analyzed using a TEM Tecnai G2 20 S-Twin from FEI. The particle size distribution was determined for a randomly selected sample of 100 NPs using the software X-ImageJ (Version: 1.52 e, winPenPack X-ImageJ Launcher from the National Institute of Health (http://rsb.info.nih.gov/ij/)). To qualitatively study the change of the particle surface over the three synthesis steps, DLS and zeta potential measurements were carried out with a Zetasizer Nano ZS from Malvern Panalytical at T = 25 °C in disposable folded capillary cells (DTS1070, Malvern Panalytical). A value of 1.4649 was used for the refractive index of the silica particles, while a refractive index of 1.3300 and a viscosity of 0.8872 cP for water, or a refractive index of 1.361 and a viscosity of 1.0400 cP for EtOH were utilized. Each measurement data was obtained by three runs with several sub-runs (backscattering angle of 173°, total time 10 min DLS, 5 min zeta potential). For comparison of the results z-average, the intensity-based harmonic mean and the polydispersity index (PDI) were used for DLS. The number-based hydrodynamic diameter (d_h,0_) of the modified SiO_2_-NPs obtained by DLS measurements were validated with NTA measurements. Therefore, high-diluted aqueous dispersions of the SiO_2_ NPs were characterized with the NanoSight LM10 system (Malvern Panalytical, Germany), equipped with a 405 nm laser, following the standards ISO19430 and ASTM E2834, using the NanoSight NTA software (Version: 3.32) to record 5 videos with 60 s and 25 fps. This allowed not only the determination of dₕ,₀ but also the particle number concentration (PNC). To validate the PNC gravimetrically, the mass fraction of SiO₂-NPs was determined for one vial from each batch. This was done by drying 0.25 mL of the NP dispersion overnight at 100 °C, also in triplicate. All measurements were conducted in triplicate at 25 °C. NTA, DLS, and zeta measurements were validated with measurements of a polystyrene particle standard (PS-ST-0.1, 107 nm, microparticles GmbH) as a reference.

### Quantification of amino functional groups on the particle surface

Prior to the functionalization of the ligand periphery of the aminated silica particles with TFMB, a potentiometric back titration was performed to determine the total amount of (de)protonatable amino FGs. Therefore, 3–5 mg of the aminated SiO_2_-NPs and SiO_2_-MPs were dispersed in 1 mM hydrochloric acid (Carl Roth GmbH) and stirred for 30 min before being separated by centrifugation at 16,000 rcf. The supernatant was collected and titrated with 1 mM sodium hydroxide solution (Carl Roth GmbH) until a neutral pH of 7 was reached. The pH values were measured using a SevenExcellence S475 pH meter (Mettler Toledo). The molar concentration of amino FGs was calculated based on the difference in HCl concentration [35]. All supernatants showed initial pH values > 3.5, ensuring particle stability during incubation. To determine the number of amino FGs on the particle surface accessible for a reaction with larger reporters molecules like the TFMB tag, a previously validated optical assay relying on the cleavable disulfide reporter *N*-succinimidyl-3-(2-pyridyldithio)propionate (SPDP) was used, that has been employed by us before for the surface FG determination of different types of surface-aminated NMs [8, 22, 36]. This SPDP assay exploits the reaction of the surface amino groups with the reporter molecule yielding an amide bond, followed by release of the photometrically detectable chromophore 2-thiopyridone (2-TP) through reductive cleavage of the disulfide bond initiated by addition of tris(2-carboxyethyl) phosphine hydrochloride (TCEP) [22, 36]. 0.2 µmol of SPDP (99%, Thermo Scientific) dissolved in 20 µL of DMSO was added to 3 mg of modified SiO_2_-NPs and SiO_2_-MPs in 480 µL of phosphate buffer (0.01 M, pH 7.4). The reaction mixtures were shaken (700 rpm) at room temperature (RT, T = 23 °C) overnight, followed by centrifugation at 15,000 rcf for 10 min. The supernatants were removed, and the particle suspensions were washed three times with 300 µL of phosphate buffer. The purified particles were redispersed in 350 µL of phosphate buffer before 50 µL (1 µmol) of TCEP (98%, Sigma-Aldrich) were added. After shaking at 700 rpm for 45 min at RT, the particles were centrifuged (15,000 rcf, 10 min) and washed twice with phosphate buffer. The cleaved amount of 2-TP in the combined supernatant fractions was determined spectrophotometrically at 343 nm (ε = 8000 L∙mol^−1^∙cm^−1^). A second optical assay was performed with the larger activatable (“turn-ON”) dye fluorescamine. This optical assay, recently automated by us, is ideal for the fast screening of surface amino FGs, in a short time under low consumption of particles to measure the reporter-accessible number of amino FG by the reporter molecule [35]. Fluorescamine, which forms an optically detectable product upon reaction with primary amino groups through a ring formation mechanism [37], is larger in size than DFMB. 15 µL (0.1 M in acetonitrile) of fluorescamine (≥ 99%, abcr GmbH) were added to 0.5 mg of surface-modified SiO_2_-NPs and SiO_2_-MPs dispersed in 1 mL of phosphate buffer (0.01 M, pH 7.9). The reaction mixtures were stirred (700 rpm) at RT for 40 min protected from light. The amount of reacted dye was quantified fluorometrically (λ_ex_ = 400 nm, λ_em_ = 482 nm). To determine the number of amino FGs a standard calibration curve using ethanolamine (0.37 nmol–15 nmol, Sigma-Aldrich) in phosphate buffer was measured.

### HR-CS-GFMAS and sample analysis

A method for fluorine determination via GaF using HR-CS-GFMAS adapted from literature [26, 27] was optimized for the analysis with the solvents EtOH (≥ 99.95%, Rotisolv®, Ultra LC-MS, Carl Roth GmbH) and DMSO (min 99.9%, p.A., ChemSolute, Th Geyer). This method consisted of a pretreatment step for sample and modifier introduction, similar to the method described by Gawor and coworkers [38]. Prior to the actual measurement, the sample and modifiers were introduced to the graphite tube, allowing for their deposition and the evaporation of the solvent. Therefore, two separate time-temperature programs were used for sample and modifier introduction and analysis, as shown in Table 1 and Table 2. Compared to the literature-known method [27], the drying steps were extended and a final temperature of 150 °C was selected to ensure complete evaporation of DMSO, which has a higher boiling point than EtOH and water. The injection of the sample and modifiers during the first step followed the procedure described by Metzger and coworkers (see Table 1) [27], while a blank (air) injection was used for the analysis method (see Table 2). In addition, three fluoride calibration approaches were used: (1) *c*(F) 0–50 µg/L in Milli-Q water, (2) *c*(F) 0–100 µg/L in 95:5 DMSO:Milli-Q water (*v/v*), and (3) *c*(F) 0–100 µg/L 95:5 EtOH:Milli-Q water (*v/v*). The fluoride solutions for the calibration curves were prepared from sodium fluoride stock solutions (1,000 mg/L; Certipur®, Merck KGaA). Samples and standards were measured in triplicate.

**Table 1:** Time temperature program for pretreatment with modifiers and sample

Step	Temperature [°C]	Ramp [°C/s]	Hold [time/s]
Drying	80	5	35
Drying	90	5	30
Drying	110	5	20
Drying	150	10	20
Pyrolysis	500	500	10
Gas adaption	500	0	5
Vaporization	500	0	6

**Table 2:** Time temperature program for analyte vaporization/GaF molecule formation

Step	Temperature [°C]	Ramp [°C/s]	Hold [time/s]
Pyrolysis	500	200	5
Gas adaption	500	0	5
Vaporization	1,550	1,500	6
Clean out	2,450	500	5

To analyze the initially aminated SiO_2_-NPs and SiO_2_-MPs, surface-modified with TFMB, the particles were dispersed in 1 mL of DMSO or 1 mL of EtOH prior to the measurement and diluted to fit into the calibration range, if necessary. Afterwards, 100 µL aliquots of each suspension were dried with N_2_ flow and redispersed in 500 µL of EtOH or 500 µL of DMSO, respectively. For quantitative analysis, daily calibration curves were prepared for each solvent.

For the determination of limits of detection (LODs) and limits of quantification (LOQs), a tenfold measurement of the blank was performed to calculate the standard deviation. The blank standard deviation was multiplied by 3 (for LOD calculation) or by 10 (for LOQ calculation) and divided by the sensitivity of the linear regression.

## Results and discussion

### Preparation and structure analytical characterization of the fluorine-labeled silica nano- and microparticles

**Fig. 1:**
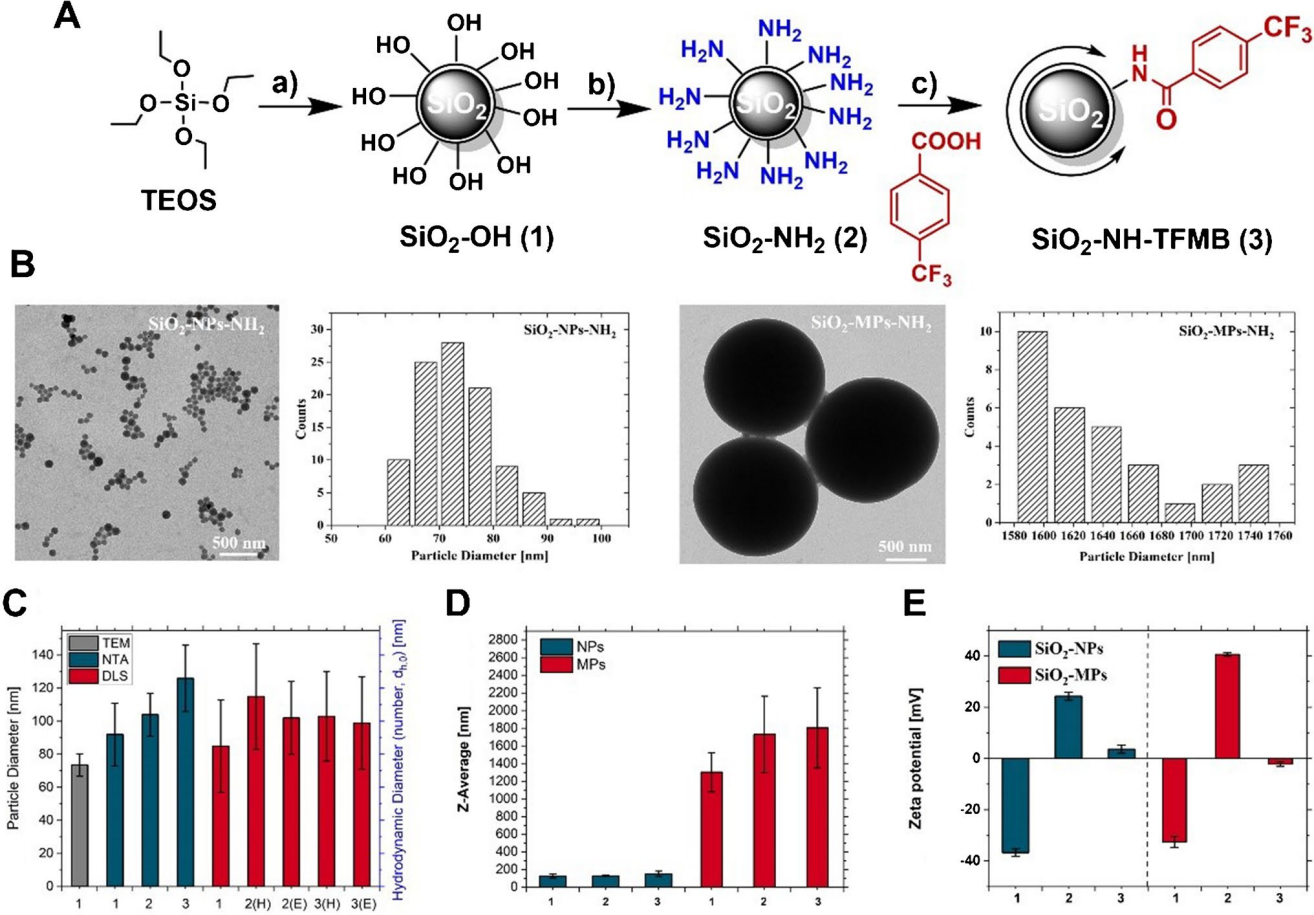
**A** Reaction scheme of the particle syntheses, also displaying the surface modification with amino groups (SiO_2_-NH_2_) followed by their labeling with a fluorine tag (SiO_2_-NH-TFMB). (a) NH_4_OH, EtOH/Milli-Q. RT, overnight; (b) EtOH, 30 °C, Ar, 48 h; (c) EDC/NHS, toluene, Ar, 40 °C, overnight; **B** TEM micrographs and histograms of SiO_2_-NPs-NH_2_ and SiO_2_-MPs-NH_2_; **C** Comparison of the obtained particle diameter (TEM) and the number-based hydrodynamic diameter (d_h,0_) obtained by DLS and NTA measurements shown for SiO_2_-NPs (E: EtOH; H: Milli-Q water); **D** Comparison of the obtained Z_average_ values for SiO_2_-NPs and SiO_2_-MPs after each reaction step measured in Milli-Q water with DLS; **E** Zeta potential measurements performed at different reaction steps for the SiO_2_-NPs and the SiO_2_-MPs

As a proof-of-concept study to assess the potential of our HR-CS-GFMAS method in conjunction with broadly available fluorine tags for the analysis of surface FGs on NPs and MPs, frequently employed in the life and material sciences, we prepared exemplarily chosen amorphous, nonporous silica particles of two different sizes bearing surface amino FGs which were then reacted with the fluorine tag TFMB. The reaction conditions were chosen to provide at least almost quantitative TFMB labeling of the surface amino FGs. The 3-step reaction scheme used for the preparation of the fluorine-modified model particles and corresponding particle characterization results are shown in Fig. 1. First, SiO_2_-NPs and SiO_2_-MPs were prepared via a modified Stöber sol-gel process, followed by a post-synthetic surface grafting with APTES to introduce amino FGs. The amount of APTES used for the reaction step was adjusted to achieve a comparable number of surface amino FGs on both particle types despite their different sizes and hence surface areas. The TEM micrographs of the resulting particles confirm the formation of spherical particles with mean particle sizes of 73.5 ± 6.8 nm and 1639.3 ± 57.0 nm. The results of DLS and NTA measurements reveal number-based hydrodynamic diameters (d_h,0_) of 85 ± 28 nm and 92 ± 19 nm for the unmodified SiO_2_-NPs, and higher d_h,0_ after amination with values of 115 ± 32 nm (DLS) and 104 ± 13 nm (NTA), respectively. Labeling of the amino surface FGs with TFMB barely affected the particle size, as revealed by DLS and NTA measurements, giving d_h,0_ of 103 ± 27 nm and 126 ± 20 nm, respectively. The aminated, TFMB-modified SiO_2_-MPs exhibited an increase of Z_average_ from 1302 ± 221 nm to 1730 ± 433 nm (SiO_2_-MP NH_2_) and 1804 ± 453 nm (SiO_2_-MP TFMB). The results of the zeta potential measurements confirm the successful covalent attachment of the amino silane, followed by the covalent binding of the fluorine tag onto the surface of both silica particles through the change of surface charge from negative to positive, and a decrease in the zeta potential after the amino FG labeling with TFMB.

**Fig. 2:**
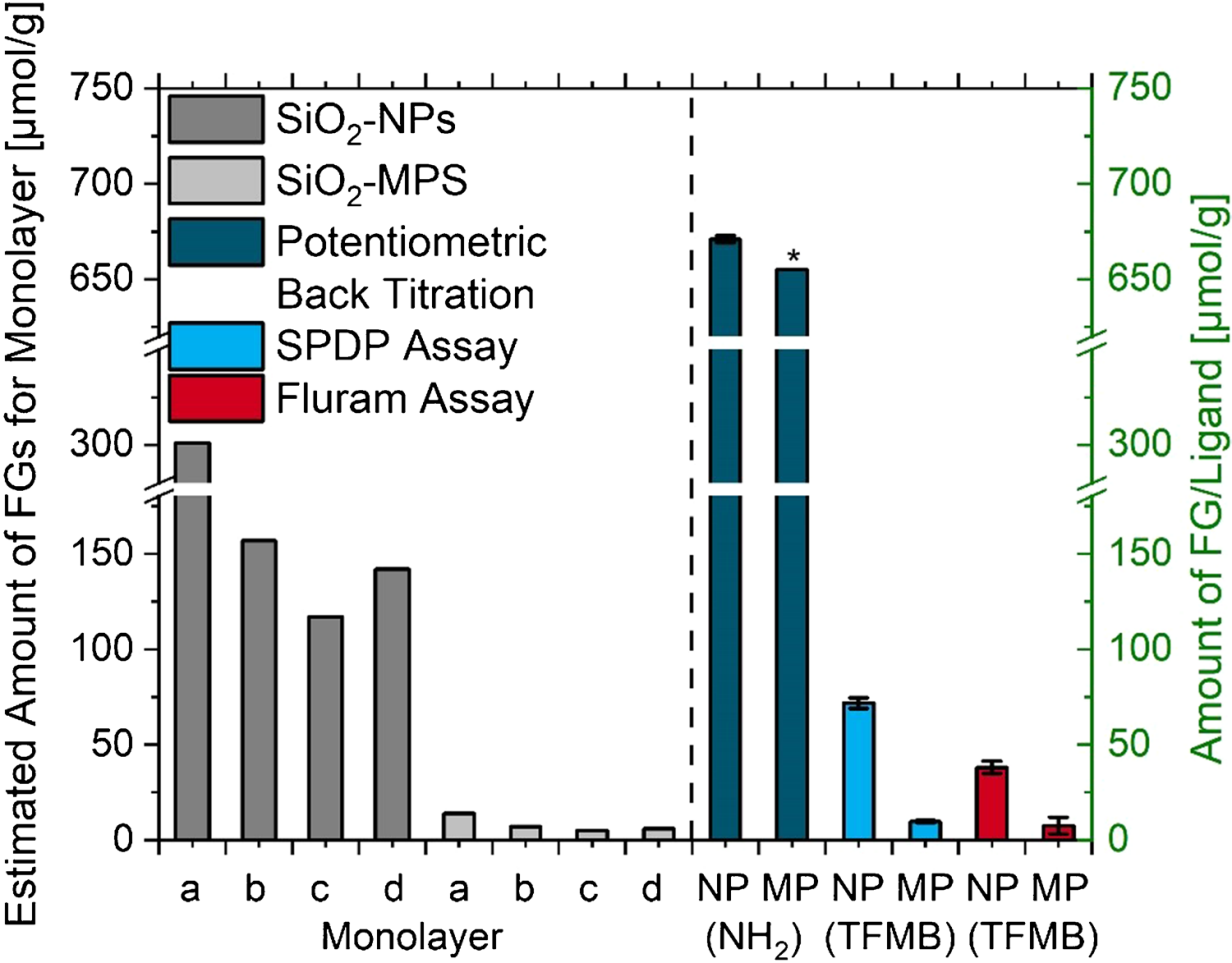
Comparison of the different methods and measurands used for the determination of the total and reporter-accessible amount of surface FGs and the estimation of the amount of reporter molecules involved in the formation of a monolayer on the surface of the aminated silica particles to estimate the number of surface amino FGs binding to the respective reporter molecules. Estimation of the amount of amino FGs (a, SiO_2_-NPs: 301 µmol/g, SiO_2_-MPs:14 µmol/g) as well as SPDP (b, SiO_2_-NPs: 157 µmol/g, SiO_2_-MPs:7 µmol/g), fluorescamine (Fluram, c, SiO_2_-NPs: 117 µmol/g, SiO_2_-MPs: 5 µmol/g), and TFMB molecules (d, SiO_2_-NPs: 142 µmol/g, SiO_2_-MPs: 6 µmol/g) per amino silane monolayer. The total amount of surface amino FGs, determined via potentiometric back titration, is represented by the dark blue bars, while the amounts of surface amino FGs obtained by the two optical assays are shown in light blue (SPDP) and red (Fluram) bars

As prerequisites to judge the reliability of our HR-CS-GFMAS method in conjunction with surface FG labeling with the tag TFMB, estimates of the amount of surface amino FGs available for the covalent attachment of TFMB were needed, thereby considering the size and spatial requirement of this label. Therefore, the specific surface areas, the amount of surface amino FGs for a monolayer of amino silane on the particle surface, and the amount of total amino surface FGs were determined or calculated. Using particle diameters and specific surface areas obtained from TEM, a silica density of 1.80 g/cm^3^, and the gravimetrically determined particle number concentration (PNC), validated for SiO₂-NPs via NTA, the amount of surface amino FGs, assuming 4 amine molecules per nm^2^ [39] per amino silane monolayer, was calculated to about 301 µmol/g (SiO_2_-NPs NH_2_) and 14 µmol/g (SiO_2_-MPs NH_2_). Following the amino silane grafting step, the maximum number of (de)protonatable amino FGs was obtained using a fast and cost-effective potentiometric back titration approach, employing ultrasmall protons as reporters [35]. Please note that this back titration method lacks, however, chemo-selectivity and cannot distinguish between different types of surface FGs. This method is also not applicable for a direct determination of the covalently bound TFMB molecules. The accordingly determined total amount of (de)protonatable groups is expected to equal the total amount of surface amino FGs for the rigorously purified aminated silica particles. As shown in Fig. 2, both SiO_2_-NPs and SiO_2_-MPs apparently exhibit a multilayer structure with a comparable amino FG coverage of about 655 µmol/g (SiO_2_-MPs, 48.5 layers) and 671 ± 2 µmol/g (SiO_2_-NPs, 2.2 layers), exceeding the amino silane monolayer estimated.

Next, to estimate the amount of surface amino FGs available for the covalent binding of TFMB, two optical assays with differently sized optical reporters were performed, which involved the covalent attachment of the respective reporter dyes to the surface amino FGs: a catch-and-release SPDP assay [22, 36] and an assay using a larger optical reporter, the "turn-ON" dye fluorescamine (Fluram assay) [35]. The estimated spatial demands of the reporter molecules are 0.48 nm^2^ and 0.66 nm^2^ for the reporters SPDP and Fluram [35], calculated here as the area of the smallest face of an oriented bounding box [22]. The calculated binding area of our fluorine tag TFMB of 0.53 nm^2^ lies in between the binding areas of these two optical reporter molecules. Figure 2, which summarizes the results derived from the potentiometric back titration and the two optical assays, underlines the influence of the different sizes, spatial requirements, and chemical interaction or binding mechanism for labeling methods. Hence, assuming quantitative amino FG labeling independent of the respective labeling chemistry used, we assume that the results obtained with the TFMB tag, read out via fluorine measurements by HR-CS-GFMAS, should lie in between the numbers provided by the two optical assays. Next, we theoretically estimated the amount of reporter molecules required to form a monolayer on the surface of the aminated silica particles to derive the coverage of the amino FGs present on the silica particle surface by the three reporters used in this study, see Fig. 2, thereby, however, neglecting possible electrostatic repulsion or other specific interactions between neighboring reporters. This yielded the binding of SPDP, Fluram, and TFMB molecules to 52%, 38%, and 47% of the total amount of surface amino FGs.

In addition, we determined the amounts of surface amino FGs before and after TFMB labeling with the smallest reporter used in this study, i.e., the SPDP assay. This provided an indirect estimate of the amount of surface amino FGs bound to TFMB molecules. The resulting numbers of 71.8 nmol/mg and 9.7 nmol/mg of bound TFMB for the aminated SiO_2_-NPs and SiO_2_-MPs correspond to 51% and 162% of the amount of theoretically estimated TFMB molecules on the SiO_2_-NPs and SiO_2_-MPs. In comparison, the analogously performed determination of the TFMB amounts with the Fluram assay done before and after TFMB functionalization yielded TFMB numbers of 38.1 nmol/mg and 7.62 nmol/mg, equaling TFMB amounts of 27% and 127%. The different results obtained with the SPDP and the Fluram assay underline the considerable influence of reporter spatial requirement for all methods involving a labeling step, as previously mentioned. We tentatively ascribe the apparently overestimated numbers derived for the aminated SiO_2_-MPs to our underlying assumption of perfectly spherical particles and the neglect of steric and electrostatic repulsion between the reporter molecules. Also, multilayer structures of amino silanes formed especially on the microparticles may contribute to this observation.

### Optimization of fluorine analysis via HR-CS-GFMAS

**Fig. 3:**
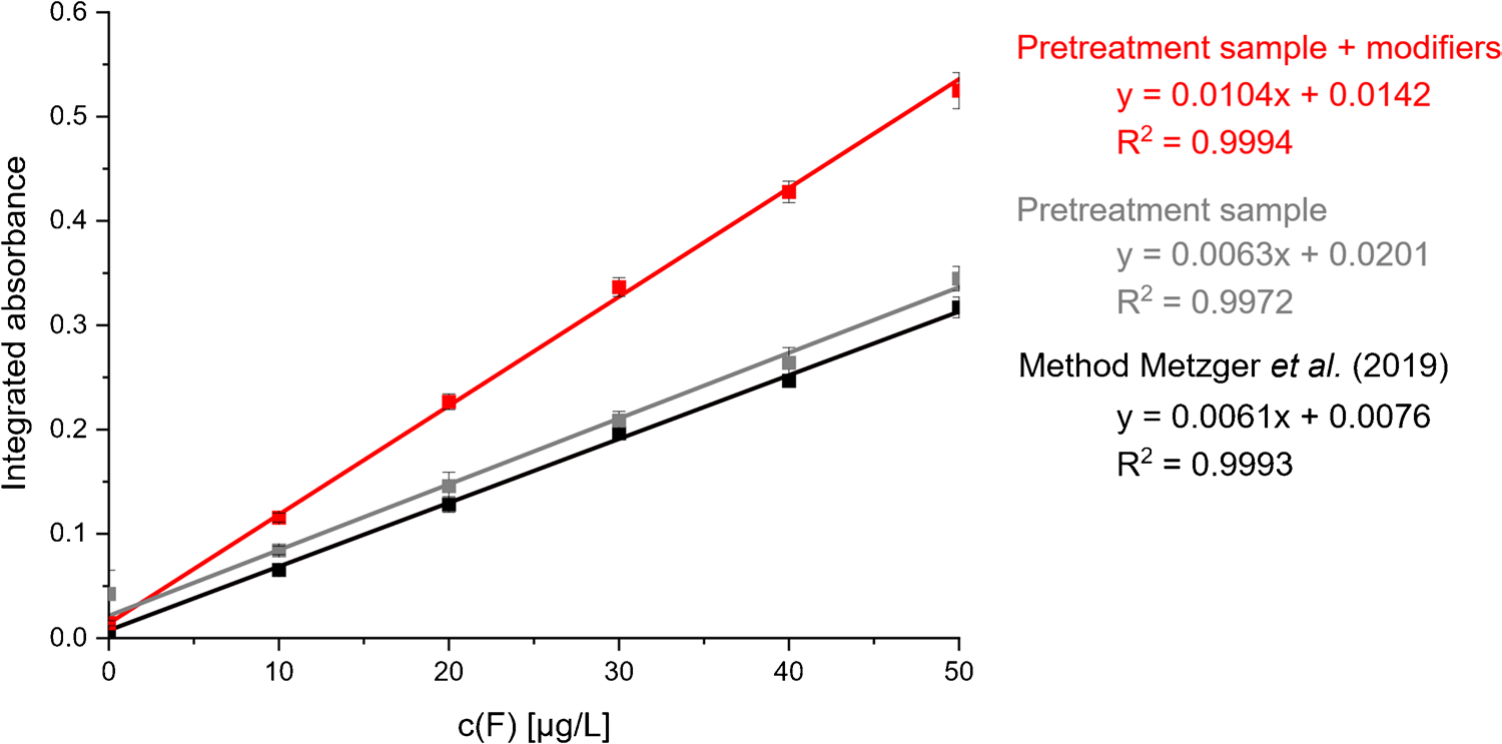
Optimization of sample and modifier introduction at the same time (method of Metzger et al*.*) (black), with pretreatment of the sample (gray) and with pretreatment of sample and modifiers (red) for fluoride (NaF) analysis in ultra-pure water using HR-CS-GFMAS via GaF detection

Next, the fluorine analysis via HR-CS-GFMAS was optimized to quantify the fluorine tags bound to the surface of the two representatively chosen and prepared NMs. First, fluoride calibrations using aqueous fluoride solution and three calibration methods shown in Table 1 and Table 2 were compared: (1) the method of Metzger and coworkers [27], (2) the introduction of the sample in a pretreatment step and modifiers in the analysis step (see Table 2), and (3) the introduction of sample and modifiers in a pretreatment step. No differences in the slopes were observed for the method by Metzger et al*.* [27] and the pretreatment with only sample introduction, that both gave integrated absorbance values of about 0.006 (Fig. 3, black, light gray). This indicates that no analyte losses occurred during pretreatment. The method which introduced both sample and modifiers in the pretreatment step showed a much higher sensitivity (Fig. 3, red). This could be associated with a deeper penetration of the Pd modifier into the graphite tube layer after pyrolysis favoring an enhanced vaporization of the analyte during the measurement [40]. Therefore, this method was selected for further studies.

**Fig. 4:**
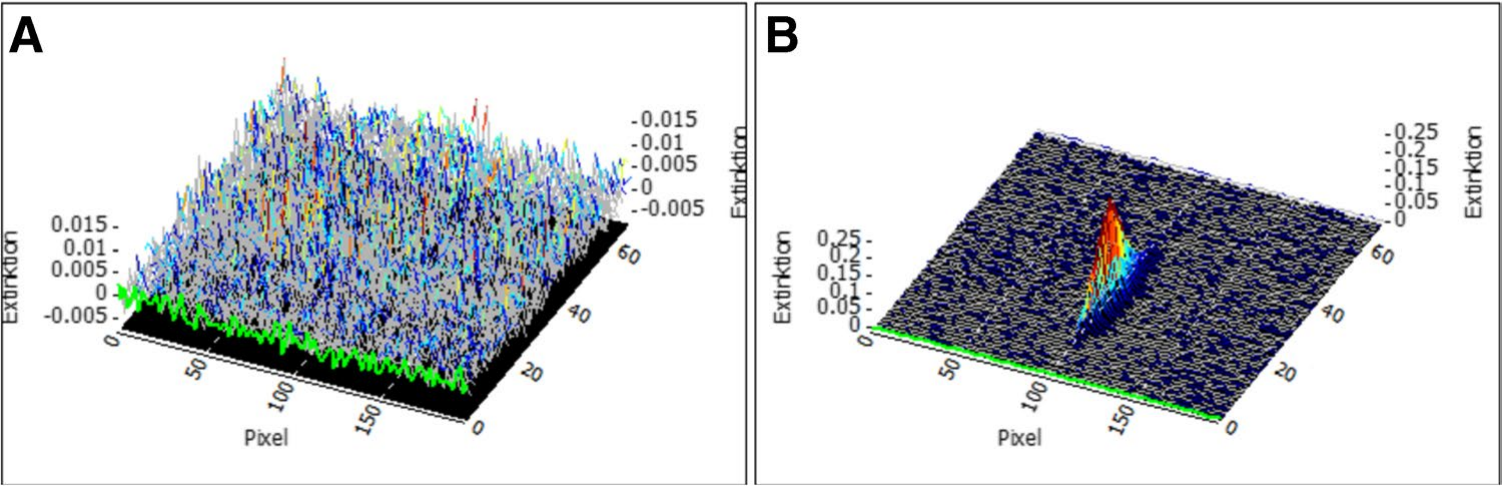
**A** Molecular absorption spectra for GaF using the method of Metzger et al*.* and **B** the newly developed method with pretreatment of the sample and modifiers for a fluoride standard solution with a concentration of *c*(F) 100 µg/L solved in DMSO

The analysis of a fluoride standard solution with a fluoride concentration *c*(F) = 100 µg/L in DMSO was compared with the results of the method from Metzger et al*.* (Fig. 4A) and the modified method based on sample and modifier pretreatment (Fig. 4B). In Fig. 4, the molecular absorption spectra of GaF are shown for both methods. The spectrum displayed in Fig. 4A reveals that no GaF absorption takes place—just blank noise is recorded (see zoom in scale). In a recent publication by Kim et al*.*, thermal decomposition of PFAS with the involvement of DMSO has been reported [41]. Thus, one possible explanation for the absence of a GaF signal could be premature decomposition/fluorine loss of the PFAS substance below the molecular formation temperature, which can then no longer be detected by HR-CS-GFMAS. Upon temperature program optimization, shown in Fig. 4B, most of the interferences which occurred during the direct analysis of the DMSO solution could be eliminated by the evaporation of the solvent during pretreatment. Thereby, a well resolved GaF peak shape was obtained.

**Fig. 5:**
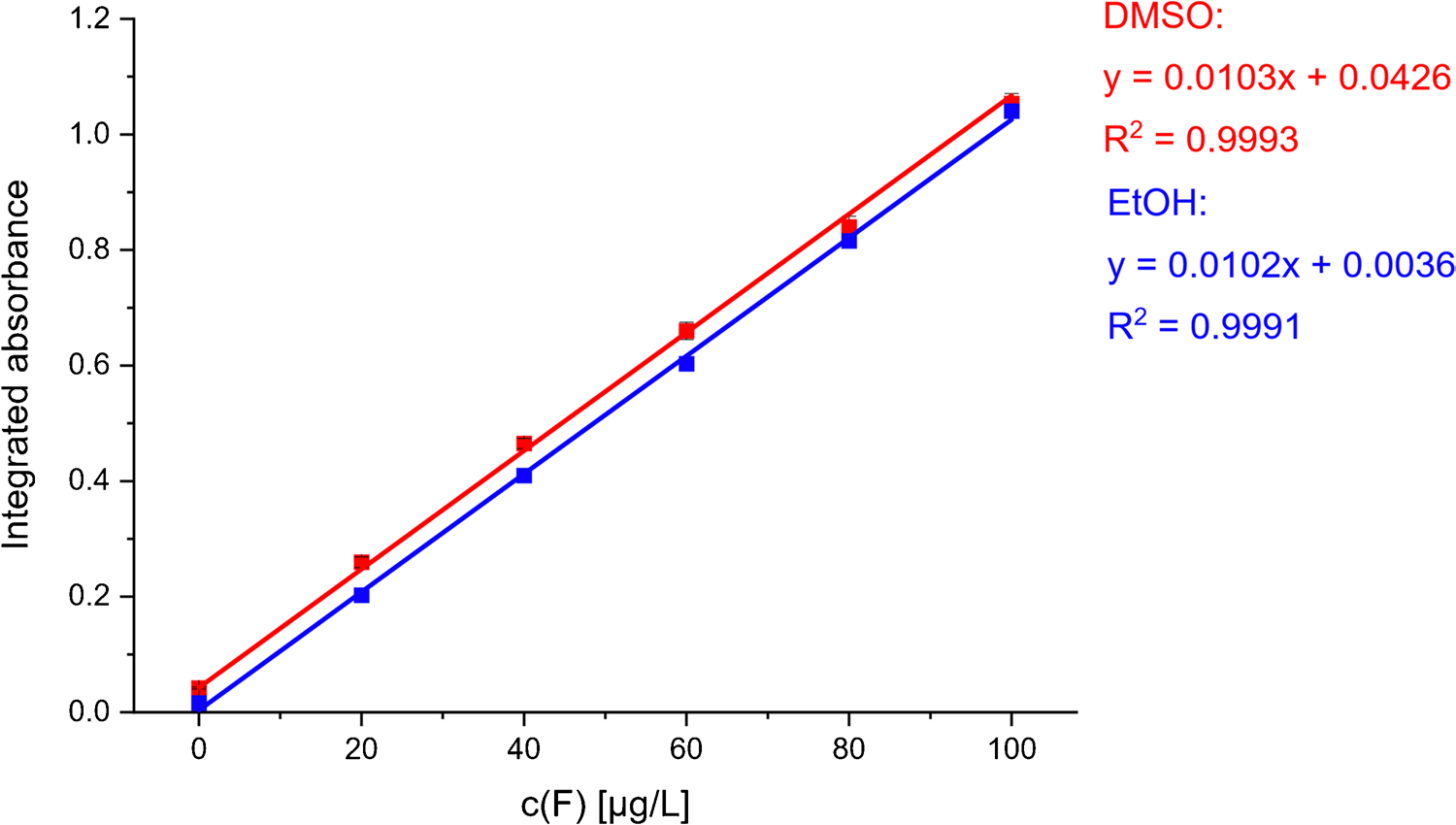
Fluoride calibration curves with c(F) 0–100 µg/L using DMSO (red) or EtOH (blue) as solvents utilizing HR-CS-GFMAS and GaF detection

In Fig. 5, fluoride calibration curves with *c*(F) 0–100 µg/L dissolved in DMSO and EtOH are compared. For the analysis using EtOH, no spectral interferences were observed for direct analysis in agreement with the results obtained with DMSO (see Fig. 4). The sensitivity obtained for fluoride samples in both solvents was very similar, with calibration curve slopes of 0.0103 (DMSO) and 0.0102 (EtOH). Furthermore, these results match well with the calibration curve slope derived from the aqueous calibration (see Fig. 3). This confirms the development of a solvent-independent calibration for fluoride analysis. However, the graphite tubes’ lifetime is reduced during analysis with DMSO solutions in comparison to EtOH or water. This could be associated with effects of corrosive compounds which could be formed at the vicinity of the graphite tube during the analysis. Potential compounds responsible for corrosive effects have not been further investigated, as this is out of the scope of this work.

LODs and LOQs for fluorine determination using either DMSO are 1.5 µg/L and 5.0 µg/L, or EtOH are 1.0 µg/L and 3.5 µg/L, respectively. Due to the higher fluorine background of the DMSO sample, the resulting LOD and LOQ exceeded those obtained for EtOH. The LODs and LOQs determined in this study are very similar to our previously reported detection (0.8 µg F/L) and quantification limits (2.7 µg F/L) in ultra-pure water [28]. These values also underpin the higher sensitivity of our AAS-based method in comparison to ^19^F NMR (LOD of 0.06 g/100 g) [42].

### Fluoride quantification of the synthesized silica nano- and microparticles

**Fig. 6:**
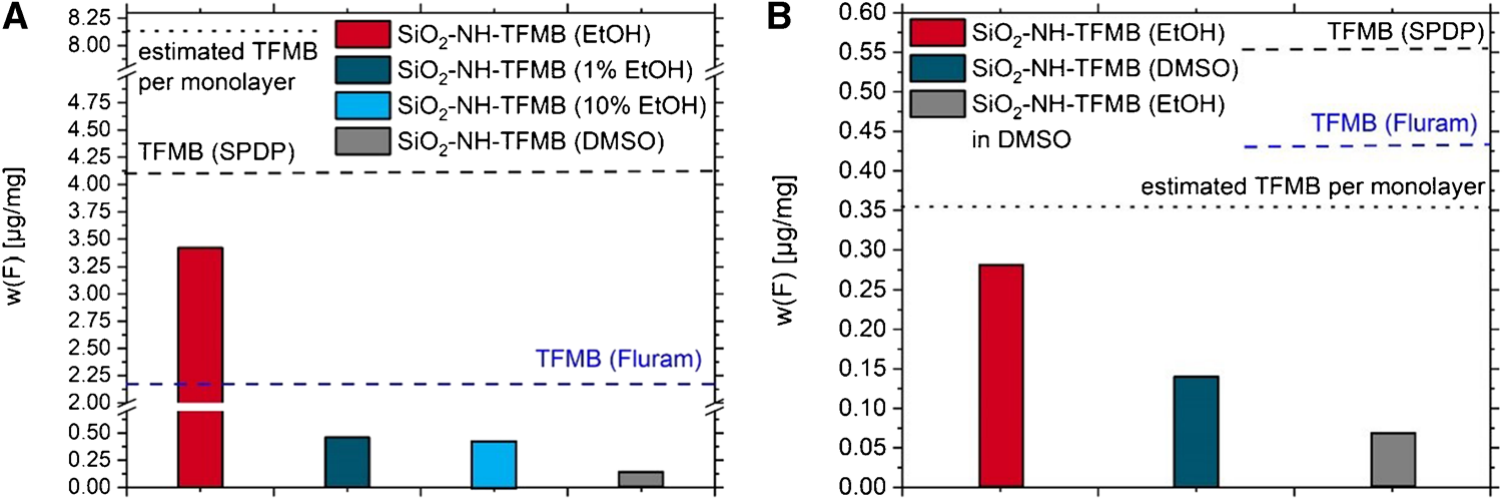
Fluorine mass fractions of **A** TFMB-labeled SiO_2_-NPs and **B** TFMB-labeled SiO_2_-MPs using DMSO and EtOH as dispersion/measurement media. These data equal mean values ± SD of triplicate measurements. The dashed blue line indicates the calculated theoretical maximum *w*(F) for the NPs and MPs

Subsequently, the fluorine content of the TFMB-labeled SiO_2_-NPs and SiO_2_-MPs was determined in DMSO and EtOH with our previously optimized HR-CS-GFMAS method. The results are shown in Fig. 6. The silica particles initially dispersed in EtOH show mean fluorine mass fractions of 3.43 µg/mg (SiO_2_-NPs) and 0.21 µg/mg (SiO_2_-MPs). These numbers are in the same order of magnitude as the amount of fluorine per mg of particles, calculated for perfectly spherical particles and a monolayer of TFMB molecules using recovery rates (compared to theoretical fluorine concentration) of 42% (SiO_2_-NPs, 8.11 µg/mg) and 58% (SiO_2_-MPs, 0.36 µg/mg), respectively. The differences between the measured amounts of fluorine/TFMB and the theoretically estimated numbers are attributed to our assumption of perfectly spherical particles and the neglect of steric and electrostatic repulsion between the reporter molecules. In addition, the quantification of surface amino FGs by potentiometric back titration indicated a multilayer structure of the amino silanes on the surface of the silica particles. The two optical assays used to indirectly determine the amount of TFMB molecules bound to amino surface FGs yielded better comparable results. The assay with the small reporter molecule SPDP provided amounts of fluorine of 4.1 µg/mg (SiO_2_-NPs) and 0.56 µg/mg (SiO_2_-MPs), while the Fluram assay with the larger Fluram molecules yielded amounts of 2.17 µg/mg (SiO_2_-NPs) and 0.43 µg/mg (SiO_2_-MPs), respectively. However, after evaporation of EtOH and redispersion of the particles in DMSO, lower fluorine mass fractions of 0.09 µg/mg and 0.05 µg/mg were obtained for the SiO_2_-NPs (see Fig. 6A) and the SiO_2_-MPs (see Fig. 6B). This suggests that either during evaporation of EtOH, redispersion in DMSO, or the HR-CS-GFMAS measurement (e.g., formation of GaF), some silica particles or fluorine analyte are lost or modified. Therefore, we also performed measurements with particles initially dispersed in DMSO. For all samples in contact with DMSO, an underestimation of the expected fluorine mass fraction was observed (see Fig. 6A and B). Since measurements with DMSO as a dispersion medium also resulted in highly resolved peaks (similar to Fig. 4B), we hypothesized that an unwanted interaction of DMSO and the fluorine tag might lead to a signal suppression or fluorine analyte loss during HR-CS-GFMAS analysis and/or modification took place.

## Conclusion and outlook

In summary, a fast, easy, and sensitive method for the screening and determination of surface amino functional groups (FGs) on nano- and microparticles with high resolution-continuum source-graphite furnace molecular absorption spectrometry (HR-CS-GFMAS) was developed, which consists of the amino FG labeling with broadly available fluorine tags, here 4-(trifluoromethyl)benzoic acid (TFMB), followed by fluorine quantification with HR-CS-GFMAS. This new method relies on the direct introduction of surface-functionalized particles, here aminated silica particles of different sizes, labeled with a fluorine tag, into the graphite furnace utilizing EtOH as dispersion medium and solvent for HR-CS-GFMAS analysis. The achieved limits of detection (LOD) and quantification (LOQ) were in the lower µg F/L range and required only small amounts of nanomaterial samples of 1–5 mg. In a first proof-of-concept study, the reliability of the HR-CS-GFMAS method involving fluorine tag labeling for the determination of the amount of surface amino FGs on silica particles was assessed by comparative measurements with electrochemical and optical methods, also requiring a reporter for signal generation. This included a potentiometric back titration, employing ultrasmall protons as reporters, and two optical assays, the SPDP and the Fluram assay, using differently sized reporter dyes, which provide the total amount of (de)protonatable surface FGs and the amount of amino FGs accessible for the reaction, i.e., labeling with the assay-specific dye reporter. The reasonably well alignment of the HR-CS-GFMAS results—given the different labeling chemistries, reporter spatial requirements, sample preparation procedures, and method sensitivities—with the results obtained with these electrochemical and optical methods provides different, yet most likely correlated measurands, confirming the applicability of our simple HR-CS-GFMAS fluorine method with the developed sample preparation, labeling, and calibration approach that exceeds, e.g., ^19^F NMR regarding sensitivity and requires only very small amounts of sample.

After a more rigorous method validation with different sets of surface-functionalized nano- and microparticles, which was beyond the scope of this first proof-of-concept study, this could pave the road for the usage of this HR-CS-GFMAS fluorine sum parameter method for process and stability control of the production of surface-functionalized nanomaterials and microparticles utilizing broadly available fluorine tags bearing different reactive groups for the labeling of common surface FGs. In this context, we currently assess the potential of our newly developed method for the analysis of other fluorine functionalized or fluorine-containing nanomaterials in different dispersion/measurement media including the further optimization for DMSO.

## Data Availability

Data are available upon request to the corresponding authors.
